# Mapping the kidney disease quality of life 36-item short form survey (KDQOL-36) to the EQ-5D-3L and the EQ-5D-5L in patients undergoing dialysis

**DOI:** 10.1007/s10198-019-01088-5

**Published:** 2019-07-23

**Authors:** Fan Yang, Carlos K. H. Wong, Nan Luo, James Piercy, Rebecca Moon, James Jackson

**Affiliations:** 1 Centre for Health Economics, University of York, York, UK; 2 Department of Family Medicine and Primary Care, Li Ka Shing Faculty of Medicine, The University of Hong Kong, Pokfulam, Hong Kong; 3 Saw Swee Hock School of Public Health, National University of Singapore, Singapore, Singapore; 4 Adelphi Real World, Bollington, UK

**Keywords:** Dialysis, EQ-5D-3L, EQ-5D-5L, KDQOL-36, Mapping, I1, C1

## Abstract

**Objectives:**

To develop algorithms mapping the Kidney Disease Quality of Life 36-Item Short Form Survey (KDQOL-36) onto the 3-level EQ-5D questionnaire (EQ-5D-3L) and the 5-level EQ-5D questionnaire (EQ-5D-5L) for patients with end-stage renal disease requiring dialysis.

**Methods:**

We used data from a cross-sectional study in Europe (France, *n* = 299; Germany, *n* = 413; Italy, *n* = 278; Spain, *n* = 225) to map onto EQ-5D-3L and data from a cross-sectional study in Singapore (*n* = 163) to map onto EQ-5D-5L. Direct mapping using linear regression, mixture beta regression and adjusted limited dependent variable mixture models (ALDVMMs) and response mapping using seemingly unrelated ordered probit models were performed. The KDQOL-36 subscale scores, i.e., physical component summary (PCS), mental component summary (MCS), three disease-specific subscales or their average, i.e., kidney disease component summary (KDCS), and age and sex were included as the explanatory variables. Predictive performance was assessed by mean absolute error (MAE) and root mean square error (RMSE) using 10-fold cross-validation.

**Results:**

Mixture models outperformed linear regression and response mapping. When mapping to EQ-5D-3L, the ALDVMM model was the best-performing one for France, Germany and Spain while beta regression was best for Italy. When mapping to EQ-5D-5L, the ALDVMM model also demonstrated the best predictive performance. Generally, models using KDQOL-36 subscale scores showed better fit than using the KDCS.

**Conclusions:**

This study adds to the growing literature suggesting the better performance of the mixture models in modelling EQ-5D and produces algorithms to map the KDQOL-36 onto EQ-5D-3L (for France, Germany, Italy, and Spain) and EQ-5D-5L (for Singapore).

**Electronic supplementary material:**

The online version of this article (10.1007/s10198-019-01088-5) contains supplementary material, which is available to authorized users.

## Introduction

The number of patients with end-stage renal disease (ESRD) is projected to increase substantially, driven by the ageing population and the rising number of people with diabetes, hypertension, and obesity [1, 2]. Due to the limited organ donors, the majority of ESRD patients have to receive maintenance dialysis and, therefore, the care for these patients has focused on improving their health-related quality of life (HRQoL) [3]. Currently, there are numerous HRQoL measures being used in patients with dialysis. In particular, the disease-specific instrument, Kidney Disease Quality of Life 36-Item Short Form Survey (KDQOL-36), is the most frequently used one in these patients and its validity and reliability has been demonstrated previously [4–6]. The KDQOL-36 has also been recommended by the United States Centers for Medicare and Medicaid to periodically collect HRQoL data for dialysis patients [7] so that the results could be used to inform and support clinicians in their decision-making and furthermore contribute to the development of clinical interventions to provide better care for dialysis patients.

KDQOL-36 consists of the Short Form 12-Item (SF-12) instrument to capture the general physical and mental well-being of the patient plus 24 items on kidney disease- and dialysis-related symptoms, effects, and burden [8]. Nevertheless, KDQOL-36 instrument is not preference-based and, therefore, does not allow the calculation of health utilities for quality-adjusted life years (QALYs) estimates. The QALYs could provide a generic health outcome comparable across disease areas and are recommended by decision makers such as the National Institute of Health and Care Excellence (NICE) [9] in their assessment of the cost-effectiveness of health care interventions. The EQ-5D is the preferred preference-based instrument to provide health utility estimates to enable QALYs calculations in the context of NICE appraisals [9]. But EQ-5D has not been a routine measure for dialysis patients and EQ-5D data of these patients is limited in literature [10]. Given the widespread use of KDQOL-36 among dialysis patients and the recommendation of using KDQOL-36 in the clinical setting, it is expected that HRQoL data measured using KDQOL-36 is accumulating. In such circumstance, the availability of a valid mapping algorithm from KDQOL-36 onto EQ-5D would make it possible to use the KDQOL-36 data in estimating health utilities for cost-effectiveness analysis (CEA). According to the Health Economics Research Centre Database of Mapping Studies [11], there is no mapping algorithm yet to map from KDQOL-36 to EQ-5D. One alternative approach is to use the currently available mapping algorithms from SF-12 onto EQ-5D [12–15], but these algorithms do not show the complete picture of KDQOL-36 (only includes 12 items of KDQOL-36) and may not produce the reliable estimates. This concern has been supported by one recently published study which reported that the EQ-5D scores mapped from SF-12 would underestimate the QALYs gained in cost-utility analysis compared to the observed EQ-5D [16], and thus there is a necessity for developing new methods to enable better health utility estimates from KDQOL-36 data for future economic evaluations in dialysis patients when EQ-5D data are not available.

Therefore, this study aimed to produce mapping algorithms from KDQOL-36 to generic EQ-5D as well as to provide a user-friendly tool for implementation.

## Methods

### Outcome measures

#### KDQOL-36

The KDQOL-36 is a 36-item self-reported questionnaire that combines the generic SF-12 instrument with disease-specific component for assessing the HRQoL of chronic kidney disease patients, adapted from the original 134-item KDQOL and the 76-item KDQOL Short Form (KDQOL-SF), with a 4-week recall period [8]. The SF-12 is the shorter version of the Short Form 36-Item (SF-36), one of the most popular generic worldwide instruments for evaluating HRQoL. It includes 12 items about general health, activity limits, ability to accomplish desired tasks, depression and anxiety, energy level, and social activities; there are 2–6 response levels for items [13]. The disease-specific component has 24 items comprising three subscales, burden of kidney disease (4 items), symptoms/problems of kidney disease (12 items), and effects of kidney disease (8 items), with 5 response levels for each item to measure how much the disease interferes with daily life and how bothered the respondent feels by symptoms/problems and the restrictions due to dialysis. The 12 items of SF-12 could be used to derive two summary measures, physical component summary (PCS) and mental component summary (MCS), ranging from 0 to 100 [17]. Responses to the three disease-specific subscales are transformed linearly to scores ranging from 0 to 100 and can be summated into the kidney disease component summary (KDCS) score [18]. As there is no overall KDQOL-36 score that incorporates all of its subscale scores, the following scores were calculated separately: PCS, MCS, burden of kidney disease (Burden), symptoms/problems of kidney disease (Symptoms), and effects of kidney disease (Effect), using the Excel file provided by the RAND Corporation [19]; and then KDCS was calculated by averaging the three disease-specific subscale scores. For all scores, higher values indicating better self-reported quality of life.

#### EQ-5D

The EQ-5D instrument has 5 items (mobility, self-care, usual activities, pain/discomfort, and anxiety/depression) [20] measuring the health on the day of survey with 3 or 5 descriptive levels for each item. In the 3-level version of EQ-5D (EQ-5D-3L), respondents choose one of three levels, ranging from ‘no problems’, ‘some/moderate problems’ to ‘unable/extreme problems’ while in the 5-level version (EQ-5D-5L), respondents choose their responses from five levels including no problems, slight problems, moderate problems, severe problems, and extreme problems. For both versions of EQ-5D, responses to the five items define a health state for which an index score can be generated to indicate its value to the general public. The index score is anchored by 0 (death) and 1 (full health), with higher scores corresponding to higher utility.

### Data

Data from two cross-sectional studies were used to develop mapping algorithms from KDQOL-36 onto EQ-5D-3L and EQ-5D-5L, respectively.

#### EQ-5D-3L

The dataset from the Adelphi CKD Disease-Specific Programme [21], a cross-sectional survey, was used to develop mapping algorithms from KDQOL-36 to EQ-5D-3L, including dialysis patients across five countries: France (*n* = 299), Germany (*n* = 413), Italy (*n* = 278), Spain (*n* = 225) and the UK (*n* = 34) [22]. The data included complete information on patients’ HRQoL measured using KDQOL-36 and EQ-5D-3L and patients’ demographic characteristics (e.g., age and sex). The country-specific EQ-5D-3L value sets [23–27] were used to calculate the EQ-5D-3L scores and then these scores were used for developing mapping algorithms for France, Germany, Italy and Spain, respectively, but not for the UK because of the small sample size (*n* = 34) [32].

#### EQ-5D-5L

Another dataset from a cross-sectional study in Singapore (*n* = 163) including patients undergoing dialysis for at least 3 months with complete data on KDQOL-36 and EQ-5D-5L was used to develop mapping algorithms onto EQ-5D-5L [28]. Patients’ socio-demographic characteristics were also available. The EQ-5D-5L value set for Singapore (unpublished data) was used to calculate the EQ-5D-5L scores. As there was no other dataset available with information on both KDQOL-36 and EQ-5D-5L, the mapping algorithms from KDQOL-36 to EQ-5D-5L were developed for Singapore only.

### Statistical analysis

#### Correlation

The estimation of a mapping algorithm relies on there being conceptual overlap between the source and the target measures [29], so the KDQOL-36 and EQ-5D are expected to be correlated. Spearman rank correlations were used to test the correlations between the KDQOL-36 subscale scores and EQ-5D index scores or item responses. The strength of correlation was defined as low, moderate, high, and very high with coefficient value of 0.30–0.49, 0.50–0.69, 0.70–0.89, and 0.90-1, respectively [30]. The correlations between the KDQOL-36 subscale scores were also tested and two highly correlated scores were not included in the same regression model. The correlations between the EQ-5D items were also explored.

#### Model development

A range of statistical models have been used in the literature for the development of mapping algorithms [11], in attempts to account for the unique distribution of EQ-5D: it is commonly skewed, multimodal, and often has one peak at 1 (indicating full health), bounded top and bottom (indicating best and worse health states) and a gap between 1 and the next feasible value. Generally, there are two broad approaches to mapping, direct mapping, which models the EQ-5D index values themselves using regression models, and indirect mapping, also referred to as response mapping, which models responses to each item of EQ-5D and then calculates the predicted utilities as a separate second step.

##### Direct mapping

Ordinary least squares (OLS) regression is the most commonly used model in direct mapping by assuming the relationship between the dependent variable (EQ-5D index values) and the independent variables can be expressed as a linear function [31]. OLS models are able to predict mean values with reasonable accuracy, but are poor at predicting those in poor health and full health [32], and the predicted values may fall outside of the plausible range.

To allow for the bounded nature of EQ-5D, mixture beta regression model could be used, as suggested by Basu and Manca [33]. This is a two-part model consisting of a multinomial logit model and a beta mixture model. It allows the estimation of dependent variables that are discrete at the bottom limit (i.e., the worst health state), at the truncation point (i.e., the second-best health state), and at the upper limit (i.e., full health), and continuous between the bottom limit and the upper limit. This method has been used in some mapping studies [34, 35] and has been shown to be more robust than OLS [36, 37].

Another mixture model which was specially developed to deal with the distributional features of EQ-5D is known as the adjusted limited dependent variable mixture model (ALDVMM) [38]. It has been shown to perform better than models used traditionally in this area [34]. It uses a mixture of adjusted normal distributions to account for the multimodality of EQ-5D by assuming that EQ-5D can be modelled as a mixture of C-components, which represent the clusters of individuals with similar utility scores. It also accounts for the peak of observations at full health and the option of a gap in the distribution below that peak, referred as truncation point as with mixture beta regression. The ALDVMM has been used with success in previous mapping studies [39–41].

##### Response mapping

In response mapping for EQ-5D, the five regression models, each for one item, together estimate the discrete distribution for all the health status in EQ-5D. The expected EQ-5D score is the average of all possible health states utilities weighted by the individual predicted probabilities. It should be noted that response mapping models require observations (preferably a sizeable number) at all levels of each item [34] and this can be a problem for EQ-5D-5L if the dataset is small and some of the item levels may not be selected by respondents. Regression models used in response mapping include multinomial logit [15], ordered logistic [42], and ordered probit [41], but these models do not account for the correlations between EQ-5D items, which may lead to biased predictions. To take the correlations into account, a recently published study applied response mapping using seemingly unrelated ordered probit models for developing mapping algorithms, although its performance was not as good as mixture models [39].

Mapping algorithms were derived in this study using all the four of the aforementioned regression methods: OLS, mixture beta regression (BETAMIX), ALDVMM, and seemingly unrelated ordered probit models (SUROPM). ALDVMM with up to three components were tested in line with methods used by the developers of the approach [38]. All analyses were undertaken in Stata using the command “*regress*” for OLS, “*betamix*” [43] for mixture beta regression, “*aldvmm*” for ALDVMM and “*cmp*” for SUROPM. The index values for the bottom limit, truncation point and upper limit were obtained from the value set specific to the country and EQ-5D version (EQ-5D-3L/EQ-5D-5L). As the country-specific EQ-5D value sets were used to calculate the EQ-5D index values, the mapping algorithms were developed for each country separately, i.e., EQ-5D-3L for France, Germany, Spain and Italy, and EQ-5D-5L for Singapore. For response mapping to EQ-5D-3L, we pooled the data of patients from the four countries together to develop the algorithms to the item responses first and then used the country-specific value sets to estimate the EQ-5D-3L index values. In supplementary analysis, we applied one value set, i.e., the UK EQ-5D-3L value set [27], to the pooled data of patients from five countries and then performed the same analyses.

As described previously, the KDQOL-36 is made up of a set of subscales scores: PCS, MCS, symptoms, effects, burden and KDCS. We mapped from the KDQOL-36 to EQ-5D using two sets of explanatory variables. First, PCS, MCS and the disease-specific summary score (i.e., KDCS) were included. Second, PCS, MCS and the disease-specific subscales scores (i.e., Symptoms, Effects and Burden) were used. We also included squared terms and all possible two-way interaction terms of KDQOL-36 subscale scores in regression models to address potential nonlinear associations. Age and sex of the patient were included in the regression models, but no other demographic or clinical covariates, to facilitate the use of the mapping algorithms to a wide range of dataset.

#### Model performance

To assess the model performance, we used the 10-fold cross-validation procedure [44]. The full sample was randomly split into 10 equally sized groups. Each combination of nine groups formed a training dataset that was used to estimate the parameters of the regression model, while the remaining group was considered as a test dataset to generate the predicted EQ-5D values based on the model developed using the training dataset. Predicted scores and observed scores were compared and mean absolute error (MAE) and root mean square error (RMSE) were calculated. This procedure was repeated until all the 10 possible training datasets were tested.

Models were ranked based on MAE and RMSE and the two rankings were summated to generate an average ranking. The model with the lowest value in average ranking would be the best-performing one [45, 46]. In the event of there being no clear difference between models, we gave priority to the model with lowest RMSE value.

All models were estimated in Stata version 15.1 (Stata Corp, College Station, TX).

## Results

### Descriptive information

**Fig. 1:**
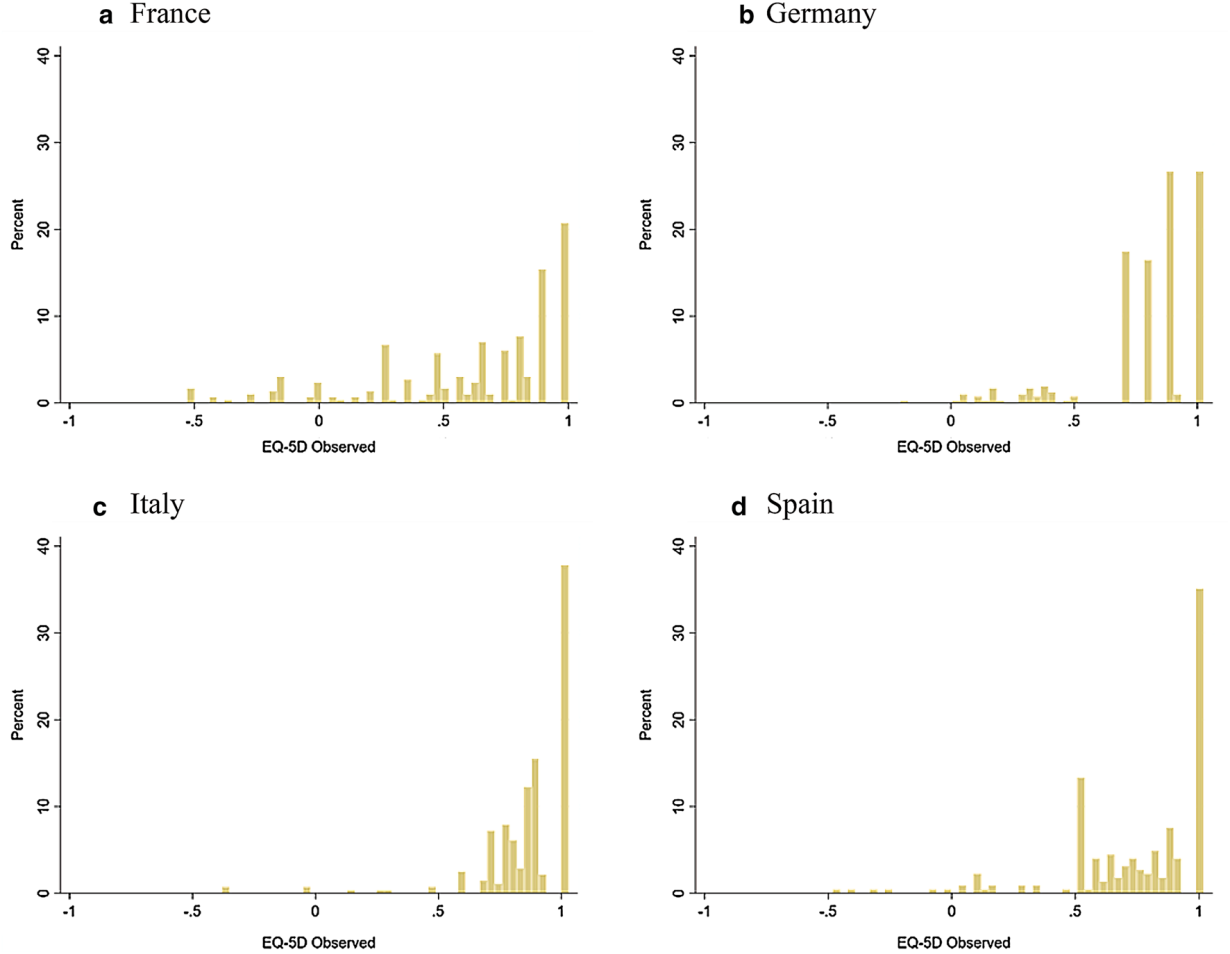
Distribution of EQ-5D-3L scores

**Fig. 2:**
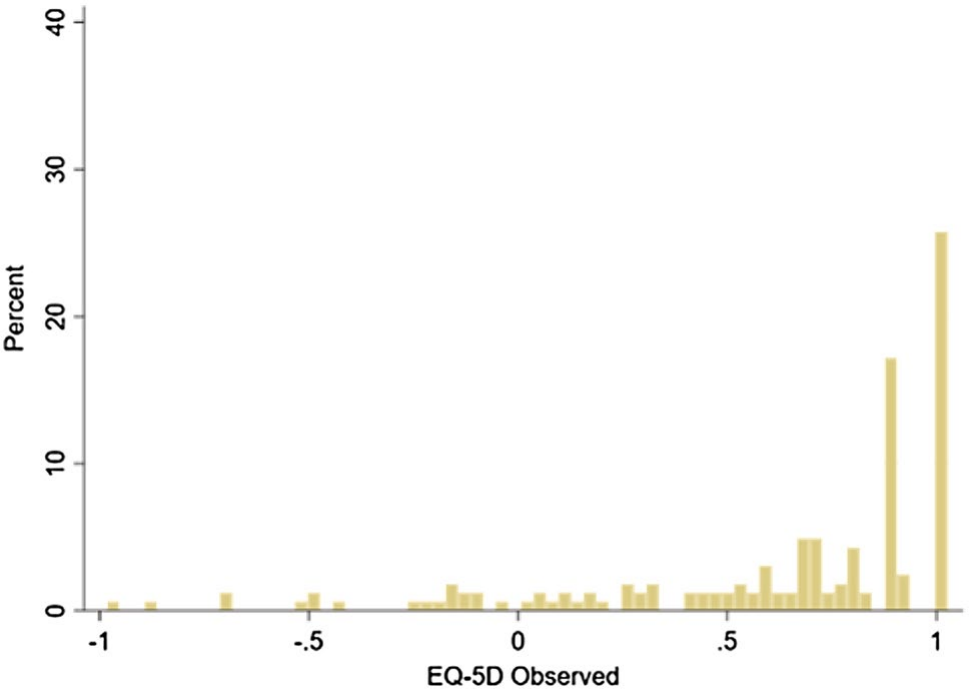
Distribution of EQ-5D-5L scores (Singapore)

Patient characteristics and summary statistics for the outcome measures are presented in Table 1. The mean age ranged from 60.5 to 66.6 years and there were more males (range 52.2%–62.5%) in all samples. The mean EQ-5D-3L score reported by patients from France, Germany, Italy and Spain was 0.622, 0.796, 0.864 and 0.746, respectively, with more than 30% patients reporting full health in Italy (37.77%) and Spain (35.11%) and about 20% in France (20.74%) and Germany (21.31%). The mean EQ-5D-5L score reported by patients from Singapore was 0.621, lower than the EQ-5D-3L scores, and 25.8% patients reported full health. Figures 1 and 2 show the distribution of EQ-5D-3L and EQ-5D-5L scores. For both EQ-5D-3L and EQ-5D-5L, the distribution is highly skewed, has a spike of observations at full health and displays the gap between full health and the next feasible state. It should be noted that the EQ-5D-3L scores had different distributions across countries (Fig. 1), which may result from the country-specific value sets and patient samples differing in health status.

**Table 1:** Health-related quality of life (HRQoL) in the samples

	EQ-5D-3L				EQ-5D-5L
	France (*n *= 299)	Germany (*n *= 413)	Italy (*n *= 278)	Spain (*n *= 225)	Singapore (*n *= 163)
	Mean (SD)	Mean (SD)	Mean (SD)	Mean (SD)	Mean (SD)
Age (years)	66.6 (14.1)	61.8 (14.4)	60.8 (13.4)	60.6 (16.4)	60.5 (11.5)
Male (%)	62.5	57.1	54.7	60.0	52.2
EQ-5D					
EQ-5D utility score	0.622 (0.383)	0.796 (0.224)	0.864 (0.185)	0.746 (0.292)	0.621 (0.447)
Proportion of EQ-5D = 1 (%)	20.74	21.31	37.77	35.11	25.8
KDQOL-36					
PCS	39.90 (10.09)	39.76 (9.33)	43.80 (10.09)	39.72 (10.92)	39.34 (9.68)
MCS	46.51 (9.61)	46.03 (10.11)	46.58 (8.06)	45.41 (10.15)	47.95 (10.83)
Symptoms^1^	78.48 (20.18)	79.50 (16.96)	86.53 (12.77)	82.24 (14.9)	80.09 (14.89)
Effects^2^	70.55 (24.45)	72.87 (18.16)	79.39 (20.25)	63.99 (22.45)	74.69 (19.43)
Burden^3^	48.20 (28.82)	54.20 (26.33)	61.15 (23.54)	55.83 (26.54)	42.45 (31.48)
KDCS^4^	65.74 (20.92)	68.86 (16.96)	75.69 (16.45)	67.36 (18.80)	65.74 (17.12)

*KDCS* kidney disease component summary, *MCS* mental component summary, *PCS* physical component summary

^1^Range 0–100, higher scores indicate that respondents are less bothered by dialysis-related symptoms (e.g., sore muscles, chest pain, cramps, etc.)

^2^Range 0–100, higher scores indicate that respondents are less bothered by the effects of kidney disease on their daily life (e.g., fluid restriction, dietary restriction, etc.)

^3^Range 0–100, higher scores indicate that to a lesser extent respondents feel kidney disease interferes with life, takes up time, causes frustration, or feels like a burden

^4^Range 0–100, average of Symptoms, Effects and Burden subscale scores

The KDQOL-36 subscale scores were similar in all five samples, but patients from Italy had higher scores in PCS, KDCS, and three disease-specific subscale scores, indicating better physical health, fewer symptoms, fewer effects on daily life and less self-perceived burden to family, consistent with the highest EQ-5D scores and more patients with full health reported by them (Table 1).

### Correlation

The Spearman rank correlation coefficients between the KDQOL-36 subscale scores and the EQ-5D index scores and items is presented in Table S1. Generally, the correlations between KDQOL-36 subscale scores and EQ-5D-3L index scores or item responses were moderate to high, although some low correlations were observed. The correlations between KDQOL-36 subscales and EQ-5D-5L index scores or item responses were low to moderate. These suggest that the two instruments overlap to some extent, which could support the attempt of mapping from one to the other.

Table S2 presents the correlations between KDQOL-36 subscale scores (excluding correlations between KDCS and the three disease-specific subscale scores, as KDCS is the average of the three scores). There was no high correlation between any two scores, so they could be included in one regression model.

The correlations between EQ-5D items were low to moderate (Table S3), supporting our approach of using seemingly unrelated regression models in response mapping, which could account for the correlations between items.

### Model

#### EQ-5D-3L

The results of the model performance mapping KDQOL-36 to EQ-5D-3L are presented in Table 2 (for France) and Table S4-S6 (for Germany, Italy and Spain).

**Table 2:** Model performance in the tenfold cross-validation for the EQ-5D-3L scores (France)

Model type	Explanatory variables included in model		Number of components		ME	MAE	RMSE	MAE rank	RMSE rank	Final rank
OLS	PCS, MCS, KDCS, age, sex	Main effect^1^	–	OLS 1	− 0.0001	0.1856	0.2437	24	22	22
		+ squared^2^	–	OLS 2	0.0005	0.1794	0.2408	18	12	14
		+ squared, interaction^3^	–	OLS 3	0.0000	0.1807	0.2435	21	21	21
	PCS, MCS, Symptoms, Effects, Burden, age, sex	Main effect	–	OLS 4	− 0.0002	0.1835	0.2441	23	23	23
		+ squared	–	OLS 5	0.0012	0.1807	0.2433	20	20	20
		+ squared, interaction	–	OLS 6	0.0000	0.1786	0.2391	17	7	13
BETAMIX	PCS, MCS, KDCS, age, sex	Main effect	–	BETA 1	0.1051	0.2067	0.2771	29	29	29
		+ squared	–	BETA 2	0.0904	0.2009	0.2716	26	26	26
		+ squared, interaction	–	BETA 3	0.0884	0.2028	0.2745	27	28	28
	PCS, MCS, Symptoms, Effects, Burden, age, sex	Main effect	–	BETA 4	0.1040	0.2102	0.2825	30	30	30
		+ squared	–	BETA 5	0.0758	0.2040	0.2728	28	27	27
		+ squared, interaction	–	BETA 6	0.0579	0.1915	0.2645	25	25	25
ALDVMM	PCS, MCS, KDCS, age, sex	Main effect	1	ALD 1-1	0.0109	0.1765	0.2385	10	5	7
			2	ALD 1-2	− 0.0003	0.1739	0.2393	5	8	5
		+ squared	1	ALD 2-1	0.0088	0.1774	0.2393	11	9	10
			2	ALD 2-2	− 0.0006	0.1780	0.2422	15	16	16
		+ squared, interaction	1	ALD 3-1	0.0083	0.1800	0.2424	19	17	19
			2	ALD 3-2	0.0003	0.1812	0.2463	22	24	24
	PCS, MCS, Symptoms, Effects, Burden, age, sex	Main effect	1	ALD 4-1	0.0103	0.1756	0.2397	7	10	8
			2	ALD 4-2	0.0012	0.1732	0.2401	3	11	6
		+ squared	1	ALD 5-1	0.0094	0.1783	0.2418	16	15	15
			2	ALD 5-2	0.0005	0.1775	0.2431	12	19	18
		+ squared, interaction	1	ALD 6-1	0.0072	0.1778	0.2389	14	6	9
			2	**ALD 6-2**	**0.0031**	**0.1730**	**0.2328**	**1**	**1**	**1**
SUROPM	PCS, MCS, KDCS, age, sex	Main effect	–	OPM 1	0.0136	0.1744	0.2382	6	4	4
		+ squared	–	OPM 2	0.0119	0.1762	0.2415	9	14	12
		+ squared, interaction	–	OPM 3	0.0107	0.1776	0.2427	13	18	17
	PCS, MCS, Symptoms, Effects, Burden, age, sex	Main effect	–	OPM 4	0.0093	0.1731	0.2339	2	2	2
		+ squared	–	OPM 5	0.0054	0.1759	0.2412	8	13	11
		+ squared, interaction	–	OPM 6	0.0076	0.1735	0.2381	4	3	3

The best-performing model is shown in bold

*ALDVMM* adjusted limited dependent variable mixture model, *KDCS* kidney disease component summary, *MAE* mean absolute error, *MCS* mental component summary, *ME* mean error, *OLS* ordinal least squares, *PCS* physical component summary, *RMSE* root mean square error, *SUROPM* seemingly unrelated ordered probit model

^1^Including PCS, MCS, KDCS, age and sex as explanatory variables

^2^Including PCS, MCS, KDCS, PCS^2^, MCS^2^, KDCS^2^, age and sex as explanatory variables

^3^Including PCS, MCS, KDCS, PCS^2^, MCS^2^, KDCS^2^, PCS*MCS, PCS*KDCS, MCS*KDCS, age and sex as explanatory variables

For France, of all the 30 models tested, RMSEs ranged from 0.2328 to 0.2825 and MAEs were between 0.1730 and 0.2102. The results of some ALDVMMs were not included if there were problems with convergence. According to MAE and RMSE, the best-performing model was ALDVMM with 2-component, which included PCS, MCS, Symptoms, Effects, Burden, and their squared terms and interaction terms, as well as age and sex as explanatory variables. For Germany, a total of 30 models were tested, among which the ADVLMM with 2-component including PCS, MCS, disease-specific subscales, age and sex as explanatory variables showed the lowest overall ranking based on MAE and RMSE. For Italy, 25 models were tested as some of the ALDVMM had problems with convergence. In contrast with the results for France and Germany, the best-performing model was the BETAMIX model including PCS, MCS, Symptoms, Effects, Burden, their squared terms, and age and sex as explanatory variables. For Spain, 28 models were tested and the best-performing one was the ALDVMM with one component including PCS, MCS, KDCS, age and sex as explanatory variables. Results of the model using the UK EQ-5D-3L value set are available in Table S7 and the best-performing model was the same one as that for France.

**Fig. 3:**
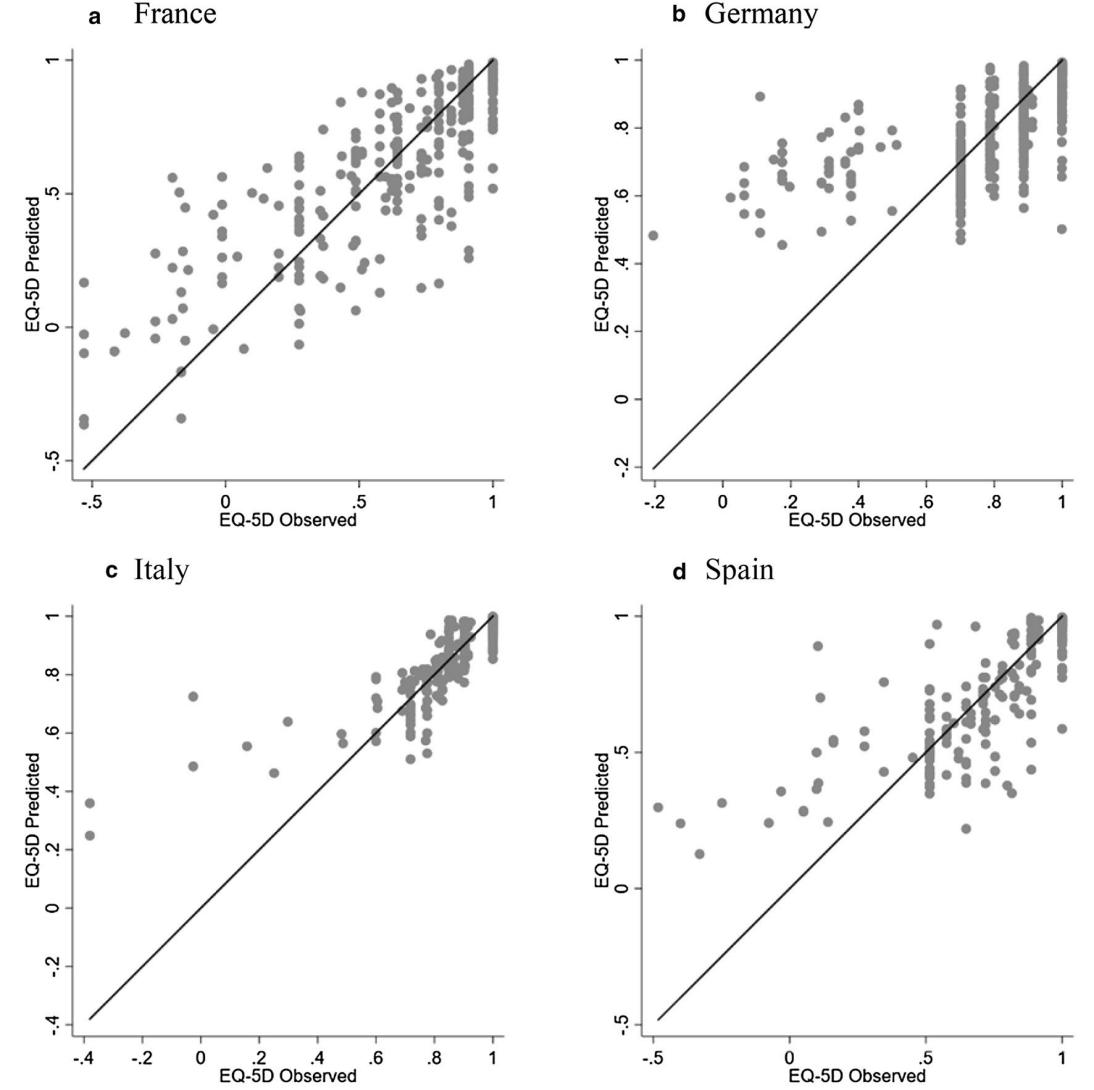
Mean predicted vs. mean observed EQ-5D-3L values using the best-performing model

Figure 3 plots mean predicted versus mean observed EQ-5D-3L values of the best-performing model for the four countries. The figures show that these mapping algorithms seem to predict well for patients at the high end of EQ-5D-3L, but may not predict very well for patients scoring at the low end of the EQ-5D-3L.

#### EQ-5D-5L

**Fig. 4:**
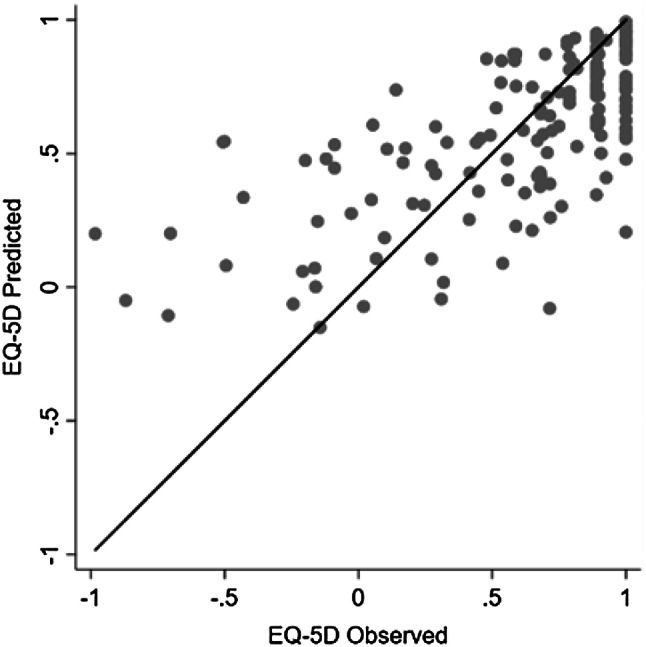
Mean predicted vs. mean observed EQ-5D-5L values using the best-performing model

The results of the model performance mapping KDQOL-36 to EQ-5D-5L are presented in Table 3. In total, 34 models were tested and the best-performing one was ALDVMM with 1-component including PCS, MCS, Symptoms, Effects, Burden, age and sex as explanatory variables. Figure 4 plots mean predicted versus mean observed EQ-5D-5L values of this best-performing model, showing that this algorithm could fit the observed data closely, but may over-predict the values when the EQ-5D observed scores were lower than 0.

**Table 3:** Model performance in the tenfold cross-validation for the EQ-5D-5L scores (Singapore)

Model type	Explanatory variables included in model		Number of components		ME	MAE	RMSE	MAE rank	RMSE rank	Final rank
OLS	PCS, MCS, KDCS, age, sex	Main effect	–	OLS 1	0.0019	0.2672	0.3503	18	11	14
		+ squared	–	OLS 2	0.0019	0.2575	0.3475	8	5	6
		+ squared, interaction	–	OLS 3	0.0012	0.2558	0.3482	5	6	4
	PCS, MCS, Symptoms, Effects, Burden, age, sex	Main effect	–	OLS 4	0.0028	0.2716	0.3511	21	12	16
		+ squared	–	OLS 5	0.0016	0.2592	0.3484	13	7	10
		+ squared, interaction	–	OLS 6	0.0185	0.2793	0.3923	26	27	27
BETAMIX	PCS, MCS, KDCS, age, sex	Main effect	–	BETA 1	0.0548	0.2714	0.3520	20	14	18
		+ squared	–	BETA 2	0.0544	0.2747	0.3584	24	18	21
		+ squared, interaction	–	BETA 3	0.0541	0.2751	0.3651	25	21	24
	PCS, MCS, Symptoms, Effects, Burden, age, sex	Main effect	–	BETA 4	0.0552	0.2667	0.3537	17	16	17
		+ squared	–	BETA 5	0.0466	0.2728	0.3738	23	22	23
		+ squared, interaction	–	BETA 6	0.0568	0.2840	0.4081	28	28	28
ALDVMM	PCS, MCS, KDCS, age, sex	Main effect	1	ALD 1-1	0.0231	0.2575	0.3421	9	2	3
			2	ALD 1-2	0.0091	0.2564	0.3487	7	8	9
			3	ALD 1-3	0.0075	0.2575	0.3522	10	15	13
		+ squared	1	ALD 2-1	0.0210	0.2583	0.3459	12	3	8
			2	ALD 2-2	0.0003	0.2552	0.3549	4	17	11
			3	ALD 2-3	− 0.0091	0.2712	0.3749	19	23	22
		+ squared, interaction	1	ALD 3-1	0.0209	0.2601	0.3499	14	9	12
			2	ALD 3-2	0.0018	0.2578	0.3620	11	19	15
	PCS, MCS, Symptoms, Effects, Burden, age, sex	Main effect	1	**ALD 4-1**	**0.0255**	**0.2558**	**0.3416**	**6**	**1**	**1**
			2	ALD 4-2	0.0034	0.2486	0.3514	1	13	7
			3	ALD 4-3	0.0151	0.2661	0.3790	16	24	20
		+ squared	1	ALD 5-1	0.0222	0.2540	0.3462	3	4	2
			2	ALD 5-2	0.0105	0.2497	0.3503	2	10	5
			3	ALD 5-3	0.0059	0.2632	0.3643	15	20	19
		+ squared, interaction	1	ALD 6-1	0.0375	0.2721	0.3848	22	25	25
			2	ALD 6-2	0.0094	0.2798	0.3895	27	26	26
SUROPM	PCS, MCS, KDCS, age, sex	Main effect	–	OPM 1	0.3637	0.4399	0.4870	31	30	31
		+ squared	–	OPM 2	0.3661	0.4444	0.4941	32	32	32
		+ squared, interaction	–	OPM 3	0.3670	0.4483	0.4990	33	33	33
	PCS, MCS, Symptoms, Effects, Burden, age, sex	Main effect	–	OPM 4	0.3642	0.4356	0.4862	30	29	29
		+ squared	–	OPM 5	0.3664	0.4356	0.4911	29	31	30
		+ squared, interaction	–	OPM 6	0.3837	0.4576	0.5214	34	34	34

The best-performing model is shown in bold

*ALDVMM* adjusted limited dependent variable mixture model, *KDCS* kidney disease component summary, *MAE* mean absolute error, *MCS* mental component summary, *ME* mean error, *OLS* ordinal least squares, *PCS* physical component summary, *RMSE* root mean square error, *SUROPM* seemingly unrelated ordered probit model

The best-performing algorithms estimated in this study can be easily implemented via Excel, which is provided in the Supplementary Materials.

## Discussion

This study aimed to develop mapping algorithms to predict EQ-5D-3L and EQ-5D-5L utility scores from the widely used KDQOL-36 instrument in the absence of directly collected EQ-5D data. By exploring different regression techniques, the algorithms using mixture models showed better predictive ability than the commonly used linear regression and response mapping models. Given the lack of previous mapping studies in this disease area and the increasing use of KDQOL-36 in the clinical setting, the mapping algorithms would provide reliable estimates for the calculation of the EQ-5D-3L and EQ-5D-5L scores as a function of KDQOL-36 and the user-friendly tool would enable researchers to implement the algorithms for EQ-5D utility values generation in applied CEA studies.

We found that the mixture models offer better model fit than linear regression and response mapping, consistent with the growing literature showing the superiority of mixture models in modelling EQ-5D [39–41]. But the target instrument in these studies was EQ-5D-3L, and our results also demonstrated the better performance of ALDVMM model in modelling EQ-5D-5L. Therefore, these findings would support the suggestion that the mixture models should be included when mapping EQ-5D from clinical measures [47], although the mapping guidelines do not recommend a specific mapping technique [48, 49].

It should be noted that the best-performing model is different in terms of regression techniques and explanatory variables for different countries and different EQ-5D versions. First, ALDVMM was the best-performing model in modelling EQ-5D-3L scores in France, Germany and Spain, but beta regression showed best fit for Italy. This may be explained by the characteristics of patients from Italy. As shown in Table 1, they reported the highest KDCS scores while the scores of patients from France, Germany and Spain were similar, and thus it is likely that they had better health than other patients did, so the mapping algorithms based on samples differing in health status were not expected to be the same. In addition, there was a strong ceiling effect of the EQ-5D-3L data for Italy (Fig. 1), which may limit the advantage of ALDVMM in addressing the multimodality of data. This may suggest the importance of considering the distribution when selecting the most appropriate model for modelling EQ-5D data, and thus future research is suggested to investigate it further. Second, the models including the KDQOL-36 disease-specific subscales scores as explanatory variables had a model fit superior to those including KDCS, but this was not the case for Spain. This may be because the three disease-specific subscale scores were highly skewed for Spain and the KDCS, which condenses them into one score, could better reflect the differences between patients, although it would discard important information. As the subscales of KDQOL-36 could be easily calculated using the Excel file provided by the instrument developer [19], the use of the KDCS is still practical to researchers populating cost-effectiveness models. Furthermore, the mapping algorithms are different when mapping to EQ-5D-3L and to EQ-5D-5L in terms of the explanatory variables included. Undoubtedly, the country-specific value set used to generate index values would contribute to the differences, but these would also be driven by the differences between the two versions of EQ-5D. The differences in the utility estimates using EQ-5D-3L and EQ-5D-5L have been reported in literature [50] and, therefore, they should not be used interchangeably. As the EQ-5D-5L is increasingly being used in practice and more EQ-5D-5L value sets are published, the results of this study suggesting the better model fit of the mixture model would help future researchers to select the appropriate model when modelling EQ-5D-5L.

This study has limitations. First, the mapping algorithms did not perform well at the low end of the EQ-5D, as illustrated in Figs. 3 and 4. This was an expected consequence of the shape of the EQ-5D distribution and the poor performance at the tails of EQ-5D distribution is a limitation common to many mapping studies [32]. Although the mixture models have been used, the impact of the distribution of EQ-5D data could not be fully addressed by the models. Second, the sample size used to derive mapping algorithms to EQ-5D-5L was small (*n* = 163). The sample size used to develop mapping algorithms should be taken into consideration when carrying out mapping [32]. This would affect the response mapping more as the models require observations at all the five levels of the EQ-5D items and a very small number of patients choosing the ‘extreme problems’ level would bias the estimation of the parameters and further limit the model performance. A larger sample would be preferable to increase the statistical power and thus lead to improved precision in estimating parameters. Third, the validity of the mapping algorithms was assessed using 10-fold cross-validation procedure. It would be preferred to assess the generalisability of the algorithms in another independent dataset; however, this was not available when conducting this study.

When such algorithms mapping the KDQOL-36 onto EQ-5D were not available, researchers who would like to generate EQ-5D values for CEAs using KDQOL-36 data have to rely on the SF-12-based functions, however, the use of these mapping functions have been found to greatly affect the QALYs estimates [16]. This study provides methods of using KDQOL-36 data to generate EQ-5D-3L and EQ-5D-5L scores. Given the requirement that the KDQOL-36 should be used in US clinical setting to assess patients’ HRQoL annually [4], it is expected that the KDQOL-36 would be more widely used in other countries to periodically collect data from patients. The algorithms developed here would provide an alternative to estimate EQ-5D from a large sample and potentially contribute to modelling the HRQoL change in CEAs assessing interventions or treatments for dialysis patients in the long-term time horizon. But it should be noted that although the mapping algorithms could provide reliable EQ-5D health utility estimates from KDQOL-36, mapping to obtain EQ-5D health utility values is still a ‘second-best’ solution [45].

## Conclusion

To the best of our knowledge, this is the first study to develop mapping algorithms from the widely used KDQOL-36 to EQ-5D-3L and EQ-5D-5L utility scores. Mapping algorithms using mixture models were found to be better than the linear regression and response mapping. A user-friendly freely accessible tool was provided to assist the implementation of these algorithms. Although it is preferred to use utilities directly derived from the EQ-5D, the algorithms can be used to generate reliable utility estimates in future economic evaluations of health care interventions for ESRD patients undergoing dialysis.

## Electronic supplementary material

Below is the link to the electronic supplementary material.

Supplementary material 1 (DOCX 56 kb)

Supplementary material 2 (XLSX 214 kb)
