# Supplementary material for: Mapping the kidney disease quality of life 36-item short form survey (KDQOL-36) to the EQ-5D-3L and the EQ-5D-5L in patients undergoing dialysis
Source: Eur J Health Econ. 2019 Jul 23;20(8):1195–206. doi: 10.1007/s10198-019-01088-5 (PMC6803593; doi:10.1007/s10198-019-01088-5)
Supplement: Supplementary file 1 — Supplementary material 1 (DOCX 56 kb) [file 10198_2019_1088_MOESM1_ESM.docx]

**Table S1.** Spearman rank correlation matrix between KDQOL-36 and EQ-5D in the training sample

|  | **EQ-5D** | | | | | |
| --- | --- | --- | --- | --- | --- | --- |
|  | **Index** | **Mobility** | **Self-care** | **Usual activities** | **Pain/discomfort** | **Depression/anxiety** |
| **France** |  |  |  |  |  |  |
| PCS | 0.72 | -0.58 | -0.50 | -0.63 | -0.58 | -0.38 |
| MCS | 0.60 | -0.42 | -0.42 | -0.54 | -0.35 | -0.54 |
| Symptoms | 0.60 | -0.42 | -0.38 | -0.46 | -0.54 | -0.48 |
| Effects | 0.57 | -0.44 | -0.41 | -0.45 | -0.48 | -0.41 |
| Burden | 0.50 | -0.39 | -0.38 | -0.39 | -0.38 | -0.43 |
| KDCS | 0.64 | -0.48 | -0.45 | -0.50 | -0.52 | -0.50 |
| **Germany** |  |  |  |  |  |  |
| PCS | 0.62 | -0.49 | -0.47 | -0.56 | -0.53 | -0.46 |
| MCS | 0.50 | -0.37 | -0.33 | -0.49 | -0.45 | -0.57 |
| Symptoms | 0.62 | -0.49 | -0.49 | -0.49 | -0.54 | -0.50 |
| Effects | 0.57 | -0.47 | -0.44 | -0.55 | -0.45 | -0.52 |
| Burden | 0.51 | -0.35 | -0.34 | -0.55 | -0.46 | -0.58 |
| KDCS | 0.67 | -0.52 | -0.50 | -0.64 | -0.57 | -0.66 |
| **Italy** |  |  |  |  |  |  |
| PCS | 0.76 | -0.67 | -0.53 | -0.68 | -0.67 | -0.44 |
| MCS | 0.36 | -0.30 | -0.36 | -0.33 | -0.22 | -0.42 |
| Symptoms | 0.71 | -0.52 | -0.41 | -0.57 | -0.63 | -0.56 |
| Effects | 0.70 | -0.50 | -0.36 | -0.53 | -0.61 | -0.55 |
| Burden | 0.65 | -0.48 | -0.35 | -0.49 | -0.54 | -0.58 |
| KDCS | 0.77 | -0.56 | -0.42 | -0.59 | -0.65 | -0.64 |
| **Spain** |  |  |  |  |  |  |
| PCS | 0.74 | -0.68 | -0.59 | -0.70 | -0.67 | -0.43 |
| MCS | 0.66 | -0.56 | -0.48 | -0.55 | -0.52 | -0.60 |
| Symptoms | 0.68 | -0.63 | -0.50 | -0.61 | -0.61 | -0.45 |
| Effects | 0.59 | -0.55 | -0.29 | -0.41 | -0.48 | -0.48 |
| Burden | 0.58 | -0.51 | -0.39 | -0.45 | -0.45 | -0.51 |
| KDCS | 0.68 | -0.62 | -0.44 | -0.54 | -0.55 | -0.54 |
| **Singapore** |  |  |  |  |  |  |
| PCS | 0.62 | -0.57 | -0.50 | -0.62 | -0.43 | -0.28 |
| MCS | 0.28 | -0.13* | -0.07* | -0.22 | -0.24 | -0.46 |
| Symptoms | 0.42 | -0.28 | -0.20 | -0.35 | -0.49 | -0.37 |
| Effects | 0.36 | -0.27 | -0.24 | -0.38 | -0.33 | -0.39 |
| Burden | 0.13 | -0.05* | -0.01* | -0.03* | -0.20 | -0.21 |
| KDCS | 0.33 | -0.21 | -0.13* | -0.27 | -0.38 | -0.39 |
| *Correlation is not statistically significant.  KDCS-kidney disease component summary, MCS-mental component summary, PCS-physical component summary | | | | | | |

**Table S2.** Spearman rank correlation matrix between KDQOL-36 scores

|  | **PCS** | **MCS** | **Symptoms** | **Effects** | **Burden** | **KDCS** |
| --- | --- | --- | --- | --- | --- | --- |
| **France** |  |  |  |  |  |  |
| PCS | - |  |  |  |  |  |
| MCS | 0.44 | - |  |  |  |  |
| Symptoms | 0.67 | 0.47 | - |  |  | - |
| Effects | 0.59 | 0.43 | 0.60 | - |  | - |
| Burden | 0.53 | 0.45 | 0.51 | 0.60 | - | - |
| KDCS | 0.68 | 0.53 | - | - | - | - |
| **Germany** |  |  |  |  |  |  |
| PCS | - |  |  |  |  |  |
| MCS | 0.46 | - |  |  |  |  |
| Symptoms | 0.64 | 0.50 | - |  |  | - |
| Effects | 0.55 | 0.50 | 0.64 | - |  | - |
| Burden | 0.46 | 0.60 | 0.45 | 0.56 | - | - |
| KDCS | 0.63 | 0.65 | - | - | - | - |
| **Italy** |  |  |  |  |  |  |
| PCS | - |  |  |  |  |  |
| MCS | 0.09 | - |  |  |  |  |
| Symptoms | 0.60 | 0.36 | - |  |  | - |
| Effects | 0.62 | 0.20 | 0.66 | - |  | - |
| Burden | 0.57 | 0.29 | 0.56 | 0.68 | - | - |
| KDCS | 0.67 | 0.31 | - | - | - | - |
| **Spain** |  |  |  |  |  |  |
| PCS | - |  |  |  |  |  |
| MCS | 0.49 | - |  |  |  |  |
| Symptoms | 0.73 | 0.56 | - |  |  | - |
| Effects | 0.54 | 0.53 | 0.64 | - |  | - |
| Burden | 0.5 | 0.58 | 0.63 | 0.69 | - | - |
| KDCS | 0.68 | 0.63 | - | - | - | - |
| **Singapore** |  |  |  |  |  |  |
| PCS | - |  |  |  |  |  |
| MCS | 0.16 | - |  |  |  |  |
| Symptoms | 0.47 | 0.35 | - |  |  | - |
| Effects | 0.46 | 0.32 | 0.63 | - |  | - |
| Burden | 0.22 | 0.36 | 0.28 | 0.34 | - | - |
| KDCS | 0.43 | 0.44 | - | - | - | - |
| KDCS-kidney disease component summary, MCS-mental component summary, PCS-physical component summary | | | | | | |

**Table S3.** Spearman rank correlation matrix between EQ-5D items

|  | **Mobility** | **Self-care** | **Usual activities** | **Pain/discomfort** | **Depression/anxiety** |
| --- | --- | --- | --- | --- | --- |
| **France** |  |  |  |  |  |
| Mobility | - |  |  |  |  |
| Self-care | 0.64 | - |  |  |  |
| Usual activities | 0.64 | 0.64 | - |  |  |
| Pain/discomfort | 0.45 | 0.41 | 0.54 | - |  |
| Depression/anxiety | 0.31 | 0.34 | 0.37 | 0.43 | - |
| **Germany** |  |  |  |  |  |
| Mobility | - |  |  |  |  |
| Self-care | 0.61 | - |  |  |  |
| Usual activities | 0.55 | 0.51 | - |  |  |
| Pain/discomfort | 0.49 | 0.39 | 0.52 | - |  |
| Depression/anxiety | 0.48 | 0.41 | 0.57 | 0.50 | - |
| **Italy** |  |  |  |  |  |
| Mobility | - |  |  |  |  |
| Self-care | 0.65 | - |  |  |  |
| Usual activities | 0.69 | 0.63 | - |  |  |
| Pain/discomfort | 0.52 | 0.43 | 0.52 | - |  |
| Depression/anxiety | 0.38 | 0.40 | 0.45 | 0.54 | - |
| **Spain** |  |  |  |  |  |
| Mobility | - |  |  |  |  |
| Self-care | 0.63 | - |  |  |  |
| Usual activities | 0.66 | 0.73 | - |  |  |
| Pain/discomfort | 0.66 | 0.46 | 0.53 | - |  |
| Depression/anxiety | 0.53 | 0.39 | 0.45 | 0.53 | - |
| **Singapore** |  |  |  |  |  |
| Mobility | - |  |  |  |  |
| Self-care | 0.56 | - |  |  |  |
| Usual activities | 0.65 | 0.66 | - |  |  |
| Pain/discomfort | 0.43 | 0.35 | 0.42 | - |  |
| Depression/anxiety | 0.35 | 0.29 | 0.38 | 0.37 | - |

**Table S4.** Model performance in the 10-fold cross-validation for the EQ-5D-3L scores (**Germany**)

| **Model type** | **Explanatory variables included in model** | | **Number of components** |  | **ME** | **MAE** | **RMSE** | **MAE rank** | **RMSE rank** | **Final rank** |
| --- | --- | --- | --- | --- | --- | --- | --- | --- | --- | --- |
| **OLS** | PCS, MCS,  KDCS,  age, sex | Main effect | - | OLS 1 | -0.0002 | 0.1232 | 0.1761 | 12 | 5 | 8 |
|  |  | + squared | - | OLS 2 | 0.0003 | 0.1227 | 0.1769 | 7 | 9 | 7 |
|  |  | + squared, interaction | - | OLS 3 | 0.0004 | 0.1239 | 0.1783 | 13 | 18 | 17 |
|  | PCS, MCS,  Symptoms, Effects, Burden,  age, sex | Main effect | - | OLS 4 | 0.0000 | 0.1225 | 0.1764 | 6 | 7 | 6 |
|  |  | + squared | - | OLS 5 | 0.0005 | 0.1232 | 0.1774 | 10 | 13 | 12 |
|  |  | + squared, interaction | - | OLS 6 | 0.0003 | 0.1258 | 0.1799 | 17 | 22 | 19 |
| **BETAMIX** | PCS, MCS,  KDCS,  age, sex | Main effect | - | BETA 1 | 0.0162 | 0.1268 | 0.1774 | 23 | 12 | 18 |
|  |  | + squared | - | BETA 2 | 0.0169 | 0.1278 | 0.1791 | 25 | 19 | 22 |
|  |  | + squared, interaction | - | BETA 3 | 0.0170 | 0.1281 | 0.1802 | 26 | 23 | 26 |
|  | PCS, MCS,  Symptoms, Effects, Burden,  age, sex | Main effect | - | BETA 4 | 0.0163 | 0.1261 | 0.1774 | 19 | 11 | 15 |
|  |  | + squared | - | BETA 5 | 0.0166 | 0.1273 | 0.1791 | 24 | 20 | 23 |
|  |  | + squared, interaction | - | BETA 6 | 0.0153 | 0.1303 | 0.1847 | 27 | 27 | 27 |
| **ALDVMM** | PCS, MCS,  KDCS,  age, sex | Main effect | 1 | ALD 1-1 | 0.0074 | 0.1228 | 0.1746 | 8 | 1 | 4 |
|  |  |  | 2 | ALD 1-2 | -0.0024 | 0.1209 | 0.1757 | 2 | 4 | 2 |
|  |  | + squared | 1 | ALD 2-1 | 0.0082 | 0.1244 | 0.1765 | 15 | 8 | 11 |
|  |  |  | 2 | ALD 2-2 | -0.0015 | 0.1209 | 0.1762 | 1 | 6 | 3 |
|  |  | + squared, interaction | 1 | ALD 3-1 | 0.0088 | 0.1254 | 0.1780 | 16 | 15 | 16 |
|  |  |  | 2 | ALD 3-2 | -0.0034 | 0.1210 | 0.1782 | 4 | 17 | 10 |
|  | PCS, MCS,  Symptoms, Effects, Burden,  age, sex | Main effect | 1 | ALD 4-1 | 0.0075 | 0.1229 | 0.1752 | 9 | 3 | 5 |
|  |  |  | 2 | **ALD 4-2** | **-0.0018** | **0.1210** | **0.1751** | **3** | **2** | **1** |
|  |  | + squared | 1 | ALD 5-1 | 0.0080 | 0.1241 | 0.1772 | 14 | 10 | 13 |
|  |  |  | 2 | ALD 5-2 | 0.0003 | 0.1232 | 0.1781 | 11 | 16 | 14 |
|  |  | + squared, interaction | 1 | ALD 6-1 | 0.0087 | 0.1265 | 0.1792 | 22 | 21 | 20 |
|  |  |  | 2 | ALD 6-2 | -0.0061 | 0.1216 | 0.1779 | 5 | 14 | 9 |
| **SUROPM** | PCS, MCS,  KDCS,  age, sex | Main effect | - | OPM 1 | 0.0047 | 0.1260 | 0.1822 | 18 | 25 | 21 |
|  |  | + squared | - | OPM 2 | 0.0081 | 0.1264 | 0.1818 | 21 | 24 | 24 |
|  |  | + squared, interaction | - | OPM 3 | 0.0053 | 0.1264 | 0.1830 | 20 | 26 | 25 |
|  | PCS, MCS,  Symptoms, Effects, Burden,  age, sex | Main effect | - | OPM 4 | -0.0034 | 0.1330 | 0.1960 | 28 | 28 | 28 |
|  |  | + squared | - | OPM 5 | 0.0008 | 0.1350 | 0.1983 | 29 | 29 | 30 |
|  |  | + squared, interaction | - | OPM 6 | 0.0019 | 0.1315 | 0.1963 | 28 | 29 | 29 |

ALDVMM-adjusted limited dependent variable mixture model, KDCS-kidney disease component summary, MAE-mean absolute error, MCS-mental component summary, ME-mean error, OLS-ordinal least squares, PCS-physical component summary, RMSE-root mean square error, SUROPM-seemingly unrelated ordered probit model

**Table S5.** Model performance in the 10-fold cross-validation for the EQ-5D-3L scores (**Italy**)

| **Model type** | **Explanatory variables included in model** | | **Number of components** |  | **ME** | **MAE** | **RMSE** | **MAE rank** | **RMSE rank** | **Final rank** |
| --- | --- | --- | --- | --- | --- | --- | --- | --- | --- | --- |
| **OLS** | PCS, MCS,  KDCS,  age, sex | Main effect | - | OLS 1 | 0.0002 | 0.0735 | 0.1169 | 14 | 17 | 16 |
|  |  | + squared | - | OLS 2 | 0.0000 | 0.0742 | 0.1169 | 16 | 16 | 18 |
|  |  | + squared, interaction | - | OLS 3 | 0.0004 | 0.0726 | 0.1140 | 11 | 8 | 8 |
|  | PCS, MCS,  Symptoms, Effects, Burden,  age, sex | Main effect | - | OLS 4 | 0.0003 | 0.0746 | 0.1184 | 17 | 20 | 20 |
|  |  | + squared | - | OLS 5 | -0.0003 | 0.0742 | 0.1158 | 15 | 14 | 15 |
|  |  | + squared, interaction | - | OLS 6 | -0.0005 | 0.0759 | 0.1176 | 19 | 19 | 21 |
| **BETAMIX** | PCS, MCS,  KDCS,  age, sex | Main effect | - | BETA 1 | -0.0023 | 0.0642 | 0.1099 | 3 | 2 | 2 |
|  |  | + squared | - | BETA 2 | -0.0021 | 0.0634 | 0.1103 | 2 | 3 | 3 |
|  |  | + squared, interaction | - | BETA 3 | -0.0017 | 0.0647 | 0.1123 | 4 | 5 | 5 |
|  | PCS, MCS,  Symptoms, Effects, Burden,  age, sex | Main effect | - | BETA 4 | -0.0020 | 0.0654 | 0.1120 | 5 | 4 | 4 |
|  |  | + squared | - | **BETA 5** | **-0.0005** | **0.0633** | **0.1078** | **1** | **1** | **1** |
|  |  | + squared, interaction | - | BETA 6 | -0.0010 | 0.0696 | 0.1210 | 6 | 21 | 14 |
| **ALDVMM** | PCS, MCS,  KDCS,  age, sex | Main effect | 1 | ALD 1-1 | 0.0052 | 0.0725 | 0.1142 | 10 | 10 | 10 |
|  |  |  | 2 | ALD 1-2 | -0.0023 | 0.0696 | 0.1139 | 7 | 7 | 6 |
|  |  | + squared | 1 | ALD 2-1 | 0.0043 | 0.0731 | 0.1163 | 12 | 15 | 13 |
|  |  |  | 2 | ALD 2-2 | - | - | - | - | - |  |
|  |  | + squared, interaction | 1 | ALD 3-1 | 0.0037 | 0.0723 | 0.1143 | 8 | 11 | 9 |
|  |  |  | 2 | ALD 3-2 | - | - | - | - | - |  |
|  | PCS, MCS,  Symptoms, Effects, Burden,  age, sex | Main effect | 1 | ALD 4-1 | 0.0054 | 0.0734 | 0.1158 | 13 | 13 | 11 |
|  |  |  | 2 | ALD 4-2 | - | - | - | - | - |  |
|  |  | + squared | 1 | ALD 5-1 | 0.0035 | 0.0724 | 0.1142 | 9 | 9 | 7 |
|  |  |  | 2 | ALD 5-2 | - | - | - | - | - |  |
|  |  | + squared, interaction | 1 | ALD 6-1 | 0.0023 | 0.0750 | 0.1175 | 18 | 18 | 19 |
|  |  |  | 2 | ALD 6-2 | - | - | - | - | - |  |
| **SUROPM** | PCS, MCS,  KDCS,  age, sex | Main effect | - | OPM 1 | 0.0015 | 0.0768 | 0.1126 | 21 | 6 | 12 |
|  |  | + squared | - | OPM 2 | 0.0003 | 0.0766 | 0.1146 | 20 | 12 | 17 |
|  |  | + squared, interaction | - | OPM 3 | 0.0069 | 0.0783 | 0.1230 | 22 | 23 | 22 |
|  | PCS, MCS,  Symptoms, Effects, Burden,  age, sex | Main effect | - | OPM 4 | -0.0028 | 0.0823 | 0.1213 | 24 | 22 | 23 |
|  |  | + squared | - | OPM 5 | -0.0045 | 0.0821 | 0.1238 | 23 | 24 | 24 |
|  |  | + squared, interaction | - | OPM 6 | -0.0038 | 0.0828 | 0.1257 | 25 | 25 | 25 |

ALDVMM-adjusted limited dependent variable mixture model, KDCS-kidney disease component summary, MAE-mean absolute error, MCS-mental component summary, ME-mean error, OLS-ordinal least squares, PCS-physical component summary, RMSE-root mean square error, SUROPM-seemingly unrelated ordered probit model

**Table S6.** Model performance in the 10-fold cross-validation for the EQ-5D-3L scores (**Spain**)

| **Model type** | **Explanatory variables included in model** | | **Number of components** |  | **ME** | **MAE** | **RMSE** | **MAE rank** | **RMSE rank** | **Final rank** |
| --- | --- | --- | --- | --- | --- | --- | --- | --- | --- | --- |
| **OLS** | PCS, MCS,  KDCS,  age, sex | Main effect | - | OLS 1 | -0.0011 | 0.1273 | 0.1887 | 15 | 13 | 13 |
|  |  | + squared | - | OLS 2 | 0.0023 | 0.1296 | 0.1885 | 18 | 12 | 15 |
|  |  | + squared, interaction | - | OLS 3 | 0.0024 | 0.1283 | 0.1896 | 16 | 15 | 16 |
|  | PCS, MCS,  Symptoms, Effects, Burden,  age, sex | Main effect | - | OLS 4 | -0.0018 | 0.1285 | 0.1896 | 17 | 16 | 17 |
|  |  | + squared | - | OLS 5 | 0.0011 | 0.1318 | 0.1899 | 21 | 17 | 19 |
|  |  | + squared, interaction | - | OLS 6 | 0.0011 | 0.1344 | 0.1966 | 22 | 21 | 21 |
| **BETAMIX** | PCS, MCS,  KDCS,  age, sex | Main effect | - | BETA 1 | 0.0055 | 0.1165 | 0.1804 | 2 | 2 | 2 |
|  |  | + squared | - | BETA 2 | 0.0080 | 0.1215 | 0.1859 | 8 | 7 | 6 |
|  |  | + squared, interaction | - | BETA 3 | 0.0080 | 0.1230 | 0.1904 | 11 | 18 | 14 |
|  | PCS, MCS,  Symptoms, Effects, Burden,  age, sex | Main effect | - | BETA 4 | 0.0047 | 0.1180 | 0.1810 | 4 | 4 | 4 |
|  |  | + squared | - | BETA 5 | 0.0065 | 0.1224 | 0.1861 | 9 | 9 | 9 |
|  |  | + squared, interaction | - | BETA 6 | 0.0063 | 0.1310 | 0.2052 | 20 | 23 | 22 |
| **ALDVMM** | PCS, MCS,  KDCS,  age, sex | Main effect | 1 | **ALD 1-1** | **0.0041** | **0.1169** | **0.1800** | **3** | **1** | **1** |
|  |  |  | 2 | ALD 1-2 | 0.0002 | 0.1157 | 0.1807 | 1 | 3 | 3 |
|  |  | + squared | 1 | ALD 2-1 | 0.0063 | 0.1225 | 0.1846 | 10 | 6 | 7 |
|  |  |  | 2 | ALD 2-2 | -0.0027 | 0.1215 | 0.1890 | 7 | 14 | 11 |
|  |  | + squared, interaction | 1 | ALD 3-1 | 0.0068 | 0.1230 | 0.1883 | 12 | 11 | 12 |
|  |  |  | 2 | ALD 3-2 | - | - | - |  |  |  |
|  | PCS, MCS,  Symptoms, Effects, Burden,  age, sex | Main effect | 1 | ALD 4-1 | 0.0032 | 0.1196 | 0.1812 | 5 | 5 | 5 |
|  |  |  | 2 | ALD 4-2 | 0.0040 | 0.1207 | 0.1877 | 6 | 10 | 8 |
|  |  | + squared | 1 | ALD 5-1 | 0.0051 | 0.1252 | 0.1861 | 13 | 8 | 10 |
|  |  |  | 2 | ALD 5-2 | -0.0007 | 0.1272 | 0.1958 | 14 | 19 | 18 |
|  |  | + squared, interaction | 1 | ALD 6-1 | 0.0054 | 0.1303 | 0.1964 | 19 | 20 | 20 |
|  |  |  | 2 | ALD 6-2 | - | - | - |  |  |  |
| **SUROPM** | PCS, MCS,  KDCS,  age, sex | Main effect | - | OPM 1 | 0.0734 | 0.1442 | 0.2058 | 23 | 24 | 24 |
|  |  | + squared | - | OPM 2 | 0.0719 | 0.1451 | 0.2038 | 24 | 22 | 23 |
|  |  | + squared, interaction | - | OPM 3 | 0.0720 | 0.1546 | 0.2139 | 28 | 28 | 28 |
|  | PCS, MCS,  Symptoms, Effects, Burden,  age, sex | Main effect | - | OPM 4 | 0.0556 | 0.1490 | 0.2061 | 26 | 25 | 25 |
|  |  | + squared | - | OPM 5 | 0.0550 | 0.1507 | 0.2068 | 27 | 27 | 27 |
|  |  | + squared, interaction | - | OPM 6 | 0.0537 | 0.1482 | 0.2063 | 25 | 26 | 26 |

ALDVMM-adjusted limited dependent variable mixture model, KDCS-kidney disease component summary, MAE-mean absolute error, MCS-mental component summary, ME-mean error, OLS-ordinal least squares, PCS-physical component summary, RMSE-root mean square error, SUROPM-seemingly unrelated ordered probit model

**Table S7.** Model performance in the 10-fold cross-validation for the EQ-5D-3L scores (UK EQ-5D-5L value set for patients from France, Germany, Italy, Spain and the UK)

| **Model type** | **Explanatory variables included in model** | | **Number of components** |  | **ME** | **MAE** | **RMSE** | **MAE rank** | **RMSE rank** | **Final rank** |
| --- | --- | --- | --- | --- | --- | --- | --- | --- | --- | --- |
| **OLS** | PCS, MCS,  KDCS,  age, sex | Main effect | - | OLS 1 | 0.0000 | 0.1490 | 0.2092 | 25 | 24 | 24 |
|  |  | + squared | - | OLS 2 | -0.0002 | 0.1452 | 0.2061 | 8 | 4 | 5 |
|  |  | + squared, interaction | - | OLS 3 | -0.0004 | 0.1443 | 0.2057 | 5 | 3 | 3 |
|  | PCS, MCS,  Symptoms, Effects, Burden,  age, sex | Main effect | - | OLS 4 | 0.0000 | 0.1482 | 0.2090 | 20 | 23 | 22 |
|  |  | + squared | - | OLS 5 | -0.0004 | 0.1453 | 0.2061 | 9 | 5 | 7 |
|  |  | + squared, interaction | - | OLS 6 | -0.0004 | 0.1454 | 0.2072 | 10 | 12 | 11 |
| **BETAMIX** | PCS, MCS,  KDCS,  age, sex | Main effect | - | BETA 1 | 0.0698 | 0.1717 | 0.2259 | 31 | 30 | 30 |
|  |  | + squared | - | BETA 2 | 0.0573 | 0.1615 | 0.2189 | 29 | 26 | 27 |
|  |  | + squared, interaction | - | BETA 3 | 0.0560 | 0.1612 | 0.2202 | 28 | 28 | 29 |
|  | PCS, MCS,  Symptoms, Effects, Burden,  age, sex | Main effect | - | BETA 4 | 0.0697 | 0.1717 | 0.2262 | 30 | 31 | 31 |
|  |  | + squared | - | BETA 5 | 0.0542 | 0.1601 | 0.2196 | 27 | 27 | 26 |
|  |  | + squared, interaction | - | BETA 6 | 0.0459 | 0.1586 | 0.2227 | 26 | 29 | 28 |
| **ALDVMM** | PCS, MCS,  KDCS,  age, sex | Main effect | 1 | ALD 1-1 | 0.0029 | 0.1482 | 0.2073 | 19 | 14 | 15 |
|  |  |  | 2 | ALD 1-2 | -0.0001 | 0.1452 | 0.2066 | 7 | 9 | 8 |
|  |  |  | 3 | ALD 1-3 | -0.0006 | 0.1446 | 0.2062 | 6 | 7 | 6 |
|  |  | + squared | 1 | ALD 2-1 | 0.0018 | 0.1473 | 0.2071 | 13 | 10 | 12 |
|  |  |  | 2 | ALD 2-2 | -0.0003 | 0.1438 | 0.2061 | 3 | 6 | 4 |
|  |  | + squared, interaction | 1 | ALD 3-1 | 0.0020 | 0.1465 | 0.2063 | 12 | 8 | 10 |
|  |  |  | 2 | ALD 3-2 | -0.0002 | 0.1443 | 0.2072 | 4 | 13 | 9 |
|  | PCS, MCS,  Symptoms, Effects, Burden,  age, sex | Main effect | 1 | ALD 4-1 | 0.0027 | 0.1481 | 0.2074 | 18 | 16 | 17 |
|  |  |  | 2 | ALD 4-2 | -0.0001 | 0.1456 | 0.2074 | 11 | 15 | 14 |
|  |  | + squared | 1 | ALD 5-1 | 0.0016 | 0.1474 | 0.2071 | 14 | 11 | 13 |
|  |  |  | 2 | ALD 5-2 | -0.0008 | 0.1428 | 0.2054 | 2 | 2 | 2 |
|  |  | + squared, interaction | 1 | ALD 6-1 | 0.0019 | 0.1476 | 0.2081 | 15 | 20 | 18 |
|  |  |  | 2 | **ALD 6-2** | **-0.0007** | **0.1414** | **0.2034** | **1** | **1** | **1** |
| **SUROPM** | PCS, MCS,  KDCS,  age, sex | Main effect | - | OPM 1 | 0.0144 | 0.1485 | 0.2081 | 22 | 19 | 21 |
|  |  | + squared | - | OPM 2 | 0.0141 | 0.1489 | 0.2088 | 23 | 22 | 23 |
|  |  | + squared, interaction | - | OPM 3 | 0.0137 | 0.1480 | 0.2076 | 16 | 17 | 16 |
|  | PCS, MCS,  Symptoms, Effects, Burden,  age, sex | Main effect | - | OPM 4 | 0.0143 | 0.1484 | 0.2078 | 21 | 18 | 20 |
|  |  | + squared | - | OPM 5 | 0.0136 | 0.1489 | 0.2097 | 24 | 25 | 25 |
|  |  | + squared, interaction | - | OPM 6 | 0.0131 | 0.1480 | 0.2086 | 17 | 21 | 19 |

ALDVMM-adjusted limited dependent variable mixture model, KDCS-kidney disease component summary, MAE-mean absolute error, MCS-mental component summary, ME-mean error, OLS-ordinal least squares, PCS-physical component summary, RMSE-root mean square error, SUROPM-seemingly unrelated ordered probit model
